# Ten steps towards integrated decision making for ecological restoration in cities: Rewilding the European beaver (Castor fiber) in Berlin, Germany

**DOI:** 10.1016/j.mex.2024.102827

**Published:** 2024-06-27

**Authors:** Sophia Rouella Edejer, Dagmar Haase, Matthew Dennis, Annegret Larsen

**Affiliations:** a Department of Geography, Lab for Urban Ecology, Humboldt University Berlin, Rudower Chaussee 16, Berlin 12489, Germany; b Department of Computational Landscape Ecology, Helmholtz Centre for Environmental Research – UFZ, Leipzig, Germany; c MCGIS, Department of Geography, University of Manchester, United Kingdom; d Environmental Sciences Group, Wageningen University & Research, Droevendaalsesteeg 3, Wageningen 6709PB, NL

**Keywords:** Multicriteria spatial decision support system, SWOT, Multispecies, Urban ecosystem, European beaver, Integrated decision making for ecological restoration in cities

## Abstract

Ensuring a livable city for all within the more-than-human discourse, restoration of urban ecosystems requires careful consideration of both human and non-human needs. However, traditional assessments and therefore most management plans usually fail to include the latter as a core planning requirement. This article presents and explains a 10-step method which simultaneously and actively considers both to identify potential restoration areas within urban ecosystems. To do so, a Strengths-Weaknesses-Opportunities-Threats (SWOT) analysis for the multispecies needs identification is combined with a Multicriteria Spatial Decision Support System (MCSDSS) for the spatial assessment. To validate this method, a case study of Berlin, Germany, an explicitly urban case, is presented. The aim of the study was to evaluate the ecosystem restoration (rewilding) potential of the city's riparian and riverine ecosystems through the enhancement of Eurasian beaver habitats.

• Method combining SWOT analysis with MCSDSS for an integrated spatial assessment
• Well-suited for multispecies (human and non-human) perspective on urban nature restoration

## Graphical abstract

**Figure:**
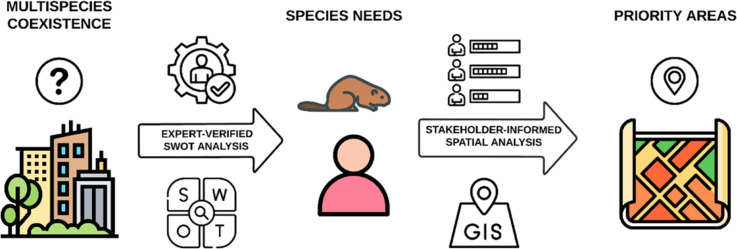

Specifications table

**Table:** 

Subject area:	
More specific subject area:	*Nature conservation, urban planning*
Name of your method:	Integrated decision making for ecological restoration in cities
Name and reference of original method:	*Treves, A., Bottero, M., Caprioli, C., & Comino, E. (2020). The reintroduction of Castor fiber in Piedmont (Italy): An integrated SWOT-spatial multicriteria based approach for the analysis of suitability scenarios. Ecological Indicators, 118(March), 106,748.*10.1016/j.ecolind.2020.106748
Resource availability:	*Not applicable*

## Background

In the face of the current ecological crisis, there's a pressing need for integrated assessments to prioritize restoration efforts, particularly in urban ecosystems where striking a balance between human and non-human needs is crucial [1,2]. However, urban planners, policy analysts, and decision-makers often neglect ecological imperatives and non-human concerns due to space and resource limitations. Yet, studies indicate that a healthy urban ecology offers various ecosystem services benefiting human physical and mental health directly or indirectly [3,4]. In the emerging discourse of multispecies coexistence, which explores the interconnectedness between humans and other species within a shared environment [5], and the necessity for sustainable, livable cities of the future, there's a compelling argument for developing new frameworks for thinking, designing, and planning.

Traditional habitat suitability models and species distribution models, while valuable, often neglect socioeconomic benefits and fail to engage in participatory decision-making [6,7]. Similarly, point process models face limitations in addressing multifunctional landscapes [8]. Although Biodiversity Sensitive Urban Design proposes a collaborative framework, its value-based approach falls short of fully capturing the potential of biodiversity-led restoration (Garrard et al., 2018; [9]). Thus, there's a need for practical guidelines to identify and address the needs of both humans and species in urban spatial decision-making.

This article presents ten steps towards integrated decision-making for ecological restoration in cities, exemplified by the rewilding of the European beaver in Berlin, Germany. Despite the promotion of the beaver's ecological role in shaping healthy waterscapes in rural landscapes, research on its potential in urban ecosystems remains limited. This study adopts a "more-than-human" perspective on urban ecosystem restoration by broadening the scope of selected evaluation criteria to include both ecological and social aspects, encompassing human and non-human nature [10].

The article suggests combining a Strengths-Weaknesses-Opportunities-Threats (SWOT) analysis for needs identification with a Multicriteria Spatial Decision Support System (MCSDSS) for spatial assessment, as proposed by Treves et al. (2019). It formalizes the overall approach and recommends its implementation in urban spatial assessments given its suitability in multifunctional contexts. The SWOT analysis provides an intuitive tool to examine human and non-human species perspectives equally. The spatial indicators for the analysis are derived from all four SWOT dimensions instead of solely on strengths and weaknesses as executed by Treves et al. (2019). In so doing, the human side of the problematic, articulated as potentials and threats from species presence, is actively included in the evaluation. The methodology does not require a particular set of indicators, making it adaptable to various local human and species compositions. The MCSDSS then facilitates strategic assessment by integrating spatial dimension with decision modeling. This enables the simultaneous consideration of conflicting objectives and interests, and the visualization of different alternatives. Stakeholder and expert involvement throughout the evaluation process accounts for preferences based on value judgments while ensuring robust scientific assumptions.

## Method details

**Fig. 1:**
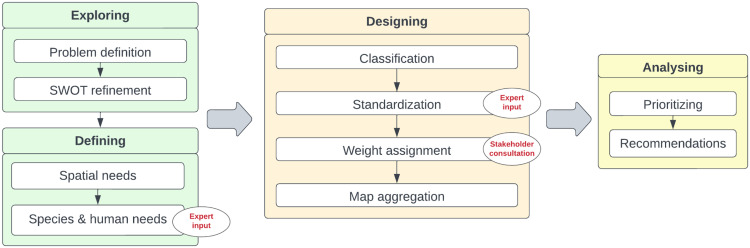
The method in 10 steps.

As illustrated in Fig. 1, the method begins by exploring and defining the structure of the problem. To conform to good scientific practices, the first step involves gaining a clear understanding of the state-of-the-art through a scientific literature review on a topic affected by land use dynamics. The aim is to identify research gaps, confirm the chosen method, and select a case study area. The following SWOT analysis describes the chosen system by eliciting the human and non-human needs involved. After defining the conceptual boundaries, the third step involves using GIS software, such as ArcGIS / ArcMap, QGIS or other geoprocessing tools available, to draw the geographic limits and characteristics of the evaluation area. In the fourth step, with the support of experts, the necessary data is gathered to evaluate the indicators described in the SWOT analysis and determine the final set of indicators for the analysis. The subsequent steps aim to represent alternative courses of action in the GIS database. Initially, the data is categorised to aid in visualising the spatial issue at hand. Standardization functions, describing indicators on a single value range, are determined through expert consultation to enable comparison. The importance of each criterion in achieving the objective is determined through questionnaires completed by stakeholders. MCSDSS usually derive weights using the pairwise comparison method according to Saaty's Analytical Hierarchy Process (AHP) [11]. Maps are aggregated to visualise spatial alternatives based on the objectives under consideration. Focus areas are then selected according to relevant criteria. Finally, recommendations for achieving multispecies (human and non-human) coexistence are made. The course of action selected will depend on the decision-making process and the specification of a decision rule. The described 10-step process provides an innovative framework for considering the presence of non-human species not solely through a species-focused lens, but with a systematic inclusion of human-related factors linked to the reduction of potential human-wildlife conflicts and the increase in ecosystem services.

### First step: problem definition

Conforming to good scientific practices, the first step consists of gaining a clear understanding of the state-of-the-art surrounding the chosen topic. This is accomplished by a thorough scientific literature review on a topic affected by land use dynamics. This method is suitable for multi-functional landscapes where trade-offs exist between human and non-human interests. It is ideal for participative processes that include preferences from a wide range of stakeholders. The selection of the target species depends on the local urban context and goals. A strategic species, such as an umbrella or keystone species, would provide the most co-benefits for a biodiversity-led restoration approach. After validating the chosen methodology, important issues are identified through a review of local scientific studies, policy and management plans, and news articles.

### Second step: SWOT refinement

The SWOT analysis categorizes information along two axes: internal or external and favorable or unfavorable to the achievement of the project objectives. In the context of multispecies coexistence, internal factors relate to the species themselves, while external factors refer to the human dimension. As factors are highly context-dependent, no specific indicators are suggested here. Instead, the following guiding questions can be raised:

- Strengths: What positive elements encourage settlement of the species?
- Weaknesses: What negative elements discourage settlement of the species?
- Opportunities: What positive elements for humans result from species settlement?
- Threats: What negative elements for humans result from species settlement?

Sources from the previous step can be helpful in answering these questions. Habitat suitability assessments can also help identify criteria that encourage or discourage species settlement. Strengths describe biophysical factors that predict species presence, while weaknesses represent human pressures on the environment. Assessments of species' ecosystem services describe the opportunities provided by the species for humans. Threats can be determined through research on human-species conflicts in local news outlets or scientific publications. Conducting interviews with local stakeholders or experts can provide the necessary precision.

### Third step: spatial needs

The first step is to select a general focus area based on the species' primary territorial needs, such as habitat type and minimum territory size, as described in the latest research under similar biophysical conditions. While rural habitat requirements are well-documented, there is still a lack of understanding of urban species behavior and their adaptations. In the absence of research, local expert interviews can provide context-specific insights.

To facilitate data aggregation and comparison between maps, it is recommended to use a standard form of data. For vector maps, a grid cell is suggested. The choice of grid geometry and size should depend on the specificities of the study. Once determined, a grid covering the entire evaluation area should be created. For raster data, a raster layer is assumed for each criterion. All layers must be processed at the same spatial resolution and projection coordinate system.

Any areas that are inaccessible or entirely unsuitable for the species' settlement are identified and excluded from the evaluation.

### Fourth step: criteria selection

The study's outcome is contingent on the quality of the available data. If funds and time permit, primary data can be collected, but this requires additional research to determine the appropriate data acquisition method. Alternatively, an exploratory data analysis can be conducted, during which raw data is evaluated based on its quality, scope, and scientific rigor. The initial data visualization and summary statistics aids in identifying possible inconsistencies, errors, outliers, or missing values within the evaluation area. If necessary, additional data can be obtained following this preliminary assessment. Indicator proxies are required when a primary indicator is unavailable or cannot be directly measured. To ensure evaluation accuracy, a suitable proxy is chosen based on scientific precedents or expert recommendations. The indicators are finalized based on the results of the data exploration. An equal number of indicators per SWOT criteria is designated to ensure a similar level of detail in the analysis of each component. Finally, expert interviews are conducted to confirm the final selection of indicators. Prior preparation of the datasets is recommended before proceeding with processing.

### Fifth step: classification

The categorization method should be based on the indicator's purpose in the evaluation and strongly supported by scientific evidence. Criteria for strengths and weaknesses, which are related to species settlement, may relate to categories that reflect the order of preference of the species as described in habitat suitability assessments. Opportunities, or ecosystem services derived from coexistence, may be classified according to the level of human need in the area. Threat categories may describe levels of risk evaluated for humans. Relevant data attributes are classified to demonstrate their distribution in the area. Factors can be expressed numerically or categorically for independent visualization and analysis, but will be converted to a standard scale in the following step.

### Sixth step: standardization

Standardization is achieved through the use of a value function, which converts the original score into a standardized value on a common scale ranging from 0 to 1, reflecting the level of suitability. Interpretation of the results differs between negative and positive elements: for strengths and opportunities, higher values indicate higher suitability, whereas for weaknesses and threats, lower values are preferred. To ensure accurate evaluation results, the standardization function must be derived from scientific literature or expert opinions in relevant fields. Public surveys can also contribute, but require careful design.

### Seventh step: weight assignment

To determine the weight of each criterion, wildlife experts/stakeholders are asked to respond to a survey. They will rank the importance of each criterion with regards to the others from the same SWOT dimension. This process is repeated for all four SWOT dimensions using a ratio scale from 1 to 9 (Saaty fundamental scale). The scale ranges from 1, representing equal influence of the two criteria in the achievement of the objective, to 9, representing the extreme importance of one criterion with respect to the other.

At this stage, it is crucial to involve various stakeholders in order to incorporate the widest possible range of preferences. Saaty's intuitive framing of the questions allows both experts and non-experts to easily contribute. However, it is important to note and control the cognitive burden of the procedure when possible [12]. The derived criteria weights represent value trade-offs made by the different actors involved and will strongly influence the final results.

When many pairs are compared, judgments are often inconsistent. To address the issue of inconsistency, Saaty introduced the Consistency Ratio (CR), the comparison between a random consistency index and a calculated consistency index (see [11] for calculation). A CR value greater than 0.1 indicates inconsistencies and requires a reconsideration of the rankings.

Once all AHP weights are sufficiently consistent (CR < 0.1), they are aggregated to produce one weight per criterion. Using the arithmetic mean of the weights derived from each respondent reflects their equal value in the evaluation process. Higher weights indicate more important parameters relative to the others in the same SWOT dimension.

### Eighth step: spatializing the SWOT: map aggregation

Aggregating all four SWOT dimensions can inform on the suitability of areas, considering the needs of both humans and other species. Combining strengths and weaknesses will result in similar rankings to a habitat suitability assessment. To assess a single ecosystem service, intersect the desired service (one opportunity indicator) with species requirements (strengths and weaknesses) and areas of conflict between humans and other species (threats).

To enhance visualisation during the decision-making process, attribute values are divided into numerical intervals of the standardised value based on their suitability, ranging from low to high.

### Ninth step: prioritizing restoration areas

After aggregating the maps, potential priority restoration areas can identified based on their ability to fulfil the study objective(s), such as proximity to the urban core, area size, etc.

### Tenth step: recommendations

Although many cities are making efforts to restore their natural areas, biodiversity is often overlooked. However, including a variety of species can greatly contribute to these efforts by increasing functionality, complexity, resilience, and cost-efficiency. Therefore, the final step of the method involves examining the existing policies and management plans of the focus areas through a multispecies lens to propose specific recommendations. These recommendations aim to increase the impact of restoration efforts while supporting multispecies coexistence.

## Method validation

**Fig. 2:**
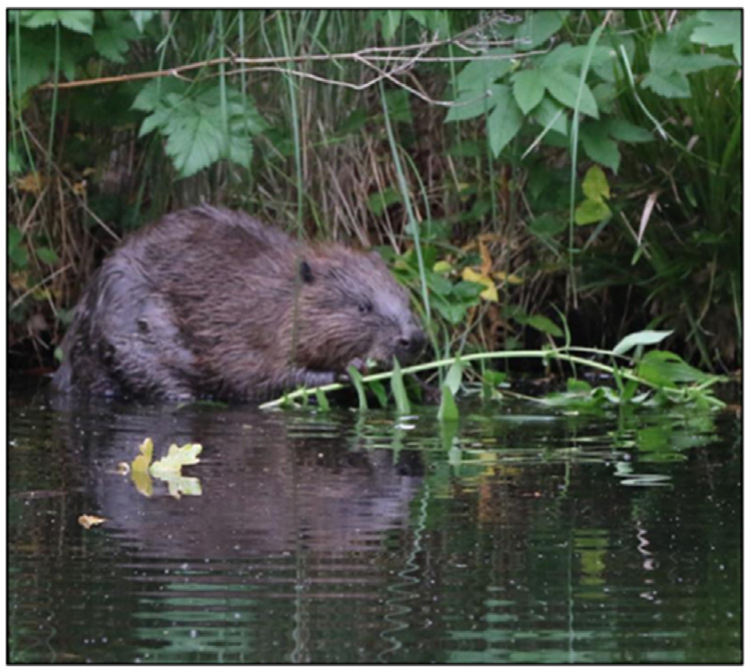
Eurasian beaver (Castor fiber) Source: Bernd Boßlet (BUND).

To validate this method, a case study of Berlin, is presented. The study aims to evaluate the potential for ecosystem restoration (rewilding) of the city's riparian and riverine ecosystems through the enhancement of Eurasian beaver habitats (refer to Fig. 2).

### First step: problem definition

With the general topic of rewilding in mind, a literature review was conducted to explore the identification of key species for rewilding efforts. Beavers were identified as one such species due to their ability to modify their surroundings as ecosystem engineers [[13], [14], [15]]. They also have a disproportionate effect on their environment relative to their abundance, notably through the creation of a mosaic of complex habitats that benefits other species as keystone species [[16], [17], [18]]. During the 19th century, habitat destruction and hunting nearly caused the extinction of beavers in Europe. Today, due to legislative and reintroduction efforts, beavers are making a comeback not only in rural areas but also in cities like Berlin. Failure to consider their needs often results in their colonization of suboptimal habitats, which can have negative impacts on humans [19,20]. As Berlin's population increases, there is a growing need to create multifunctional natural landscapes for all residents. The premise is that beavers can restore urban landscapes and provide essential ecosystem services if given the space.

The ultimate aim of this study was to evaluate the rewilding potential of Berlin's riparian and riverine ecosystems. To achieve this, three sub-objectives were identified based on the city's unique challenges: conserving beavers, mitigating the Urban Heat Island (UHI) by increasing local water absorption, and increasing rewilded urban green and blue spaces (UGBS).

### Second step: SWOT refinement

The study analyses the positive and negative elements influencing beaver settlement, categorised as strengths and weaknesses respectively. Additionally, it examines the positive effects, referred to as opportunities, and negative effects, referred to as threats, of beaver settlement for humans as presented in Table 1. Although many positive impacts of beaver settlement are dependent on damming, benefits related to digging, selective herbivory or simply the presence of beavers should not be ignored.

**Table 1:** Variables resulting from the SWOT analysis.

Strengths	Sources	Weaknesses	Sources
Presence of suitable food	[[21], [22], [23], [24], [25], [26]]	Human constructions on water	Expert interviews & local news: [27,28]
Perennial water	[[22], [24],^29^]	Bank walls and reinforcements	Expert interviews & [23]
Watercourse with reduced slope	[24]	Proximity to rail and road network	[23]
Deep water	[29]	Human disturbance on water	[23,30]
Adequate bank substrate	[29,30]	Human disturbance on land	[24,[26], [30]]
Presence of wetlands	[26]	Populated area	[21]
Presence of protected area	[30]	Traps, intentional and unintentional	[31]
Deep and steep banks (for burrowing)	[23,29]		

Opportunities	Sources	Threats	Sources

River restoration/rewilding	[[14], [32]]	Modification of cultural landscapes	Expert interview
Increase in groundwater recharge	[33,34]	Uncontrolled flooding	([35]) & local news: [36]
Mitigation of droughts and forest fire risk	[[33], [37]]	Degradation and destabilization of banks	[38]
Mitigation of floods	[[33], [39],^40^]	Erosion	[[38], [41]]
Regulation of water temperature	Dittbrenner et al. (2022)	Crop damages	[[40], [41]]
Regulation of air temperature	Effect of wetlands: [42]	Infrastructure damage	[30]
Creation of rewilded UGBS	[43]	Promotion of non-native species	[44]
Improvement of water quality	[[33], [45],^46^]	Die-off of certain species	[47]
Promotion of biodiversity	(Law et al., 2014; [[17], [40],^48^,^49^])	Threat to protected species which could be endangered by beavers	Expert interviews & [50]
Promotion of riparian forest and deadwood dynamics	[[18], [50]]	Tree felling	([[35], [41]]) & local news: [19,[20], [51]]
Influence on carbon cycle	[[33], [52]]	Disturbance of human engineering, e.g. pipes, culverts	[35]
Influence on nutrient cycling	[[15], [45]]	Barrier to fish movement	[53]
Tourism and recreation	[[45], [54]]		
Wellbeing and health	[45,55]		

### Third step: spatial needs

The semi-aquatic beaver requires the presence of perennial water, meaning that only areas surrounding water bodies and rivers, moors, and swamps were considered. The animal needs a minimum of 1 km of shoreline, up to 5 km depending on habitat suitability [56]. Therefore, lakes or ponds with a perimeter under 1 km were excluded. The lower limit was chosen because urban beavers have demonstrated great adaptability in terms of territory size [57,58].

As central-place foragers, beavers only travel a limited distance from their lodge to gather food or building materials. Many studies on habitability use a short maximum foraging distance [[21], [24],^29^]. A study was conducted to investigate the differences in beavers' foraging preferences between urban and rural riparian forests in Saskatchewan. The results indicated that urban beavers traveled further away from the water's edge due to the lack of suitable food [44]. An expert interviewee suggested that a similar situation could occur in Berlin due to the lower density of trees. To ensure objectivity, a 100 m buffer on both sides of Berlin's waterbodies has been defined as the limits of the core evaluation area, as seen in other studies [59,60].

As most of the data for Berlin was available as shapefiles, vector maps were produced using a hexagonal grid to provide a comparable basis between the different indicators. When considering connectivity and movement, hexagons are considered the most suitable grid geometry [61]. Each hexagon covered approximately 1 hectare, with edge lengths of 62.04 metres. This was considered precise enough to cover the entire area of the State of Berlin. According to the Deutsche Wildtier Stiftung (n.d.), beavers require at least 1 km of shoreline. Therefore, a minimum of 10 connected hexagons is necessary to represent their habitat territory in larger rivers. For smaller streams where both banks are contained in one hexagon, a minimum of 5 connected hexagons is sufficient.

For the study, unsuitable areas for beavers included completely built-up or fenced-off areas. These areas were removed from the hexagonal grid encompassing all of Berlin. Only hexagons within or intersecting the buffer area were kept. Hexagons outside of Berlin's administrative borders were removed, as well. The remaining hexagons were assigned a unique numerical ID and effectively covered approximately 4026 hectares of Berlin's shoreline.

### Fourth step: criteria selection

The main data provider was the State of Berlin, which regularly collects and publishes datasets on environmental and socio-economic dimensions of the city on its geo portal, FIS-Broker (https://fbinter.stadt-berlin.de/fb/index.jsp), as web-map-features. Notably, datasets from the Environmental Atlas (https://www.berlin.de/umweltatlas/en/), and ATKIS, the Authoritative Topographic-Cartographical Information System, were used and analysed in this study (refer to Table A2 (Annex) for more information). Two experts assisted in the selection of twelve criteria, three per SWOT dimension, based on their relevance to the Berlin context and the availability of suitable proxies.

Strengths include three biophysical indicators: Presence of suitable winter food (S1), an appropriate water level (S2) and a natural water network (S3). The first two criteria are present in most beaver habitat suitability evaluations. The third criterion represents an intentional adaptation to an urban context. Indeed, unlike rural waters, most urban systems are strongly altered, affecting water morphology and dynamics, which in turn affect beaver activities.

The main anthropogenic obstacles to beaver settlement are captured by the weaknesses, which include human activity (W1), human presence at night (W2), and the risk of road accidents (W3). The first criterion considers the land-use of all open and green spaces, capturing the beaver's ability to colonise different urban spaces. Due to the nocturnal behaviour of beavers, the second weakness criterion considers not only the type of disturbance, but also the period and time of disturbance [23]. The final criterion is based on local statistics identifying roadkill as the primary cause of beaver deaths [62].

The opportunities are described as follows: the creation of rewilded areas (O1), the effect on the local water balance (O2), and the effect on local cooling (O3). The selected synergies are based on the premise that biodiversity-rich functional ecosystems can help address key urban issues, such as the low supply and lack of diversity of UGBS, reduced groundwater recharge, and increasingly high urban temperatures in the summer. Given the absence of qualitative data on UGBS, O1 is predicted based on the supply of urban green spaces and is intended as a reasonable rapid assessment.

Finally, the physical threats that have been evaluated include uncontrolled flooding (T1), the risk of damage due to tree felling (T2), and bank destabilization (T3). In an urban context with narrow channels and high infrastructure density, the first criterion captures the risk of beaver dams or blocked culverts causing flooding and property damage and is predicted based on data on potential flood zones resulting from increased rainfall. This proxy is the closest available predictor without extensive measurement of hydrological dynamics and is therefore suitable for assessing current and future potential risks. The second criterion represents the danger of half-felled trees to passers-by and private and public property. Finally, beavers burrowing into non-concreted banks up to 4 metres deep can destabilise them, posing a significant risk to paths or roads whose structural integrity may be compromised [35], potentially leading to injuries or damage to public and private infrastructure.

### Fifth step: classification

Some datasets were already classified informatively, while others required manual classification based on data entries. For example, S1 required the classification of data into relevant tree genera categories. For W2, hexagons were classified based on a total score that combined both the distance and amount of artificial lighting. A higher score reflected closer and/or more artificial lighting.

### Sixth step: standardization

Once data was classified, a standardization function was applied, with the support of experts. In this study, the function depended on the type of data: Boolean, continuous, discrete with a precedent or discrete with no precedent. For the first characteristic, the data could take a value of either 0 (absence) or 1 (presence). Regarding the second characteristic, the data was normalized. As for the third characteristic, the function was adapted from literature to account for the specific urban context of Berlin, as described for W1 in Table A1 (Annex). Lastly, when no precedent in literature existed, such as for S3, the standardization function simply reflects gradual equal intervals from the least suitable to the most suitable, as recommended by interviewed experts.

### Seventh step: weight assignment

Table 2 displays the individual and mean weight, as well as the standard deviation (SD), derived from the replies of the four experts (A–D) for each of the twelve criteria. Three out of four experts strongly ranked the presence of suitable winter food as the most important factor for beaver settlement, with a mean weight of 0.62. This was followed by the natural water network with a weight of 0.23, and finally water depth with 0.15. In terms of weaknesses, the weight is more equally distributed between the three dimensions, with the risk of road accidents ranking the highest at 0.4, followed by human activity and human presence at night, both scoring 0.3. Therefore, all three dimensions are considered almost equally important in negatively affecting beaver settlement, with a slightly stronger effect of the first dimension. Regarding opportunities, the creation of rewilded areas is by far the most important criterion for three out of four experts, with a mean weight of 0.61. This is followed by the effect on local absorption with 0.29 and the effect on local cooling with 0.11. Finally, the three dimensions of threat are given equal weight, with uncontrolled flooding at 0.37 being slightly more influential than the risk of bank destabilization at 0.35, followed by the risk of tree felling at 0.28.

**Table 2:** Weight assignment for all criteria.

#	Criteria	A	B	C	D	Mean	SD
S1	Presence of suitable winter food	0.70	0.77	0.80	0.20	0.62	0.28
S2	Water depth	0.09	0.13	0.08	0.31	0.15	0.11
S3	Natural Water Network	0.21	0.11	0.12	0.49	0.23	0.18
W1	Human activity	0.06	0.67	0.29	0.17	0.30	0.26
W2	Human Presence at night	0.29	0.09	0.65	0.17	0.30	0.25
W3	Risk of road accidents	0.65	0.24	0.06	0.67	0.40	0.30
O1	Creation of rewilded areas	0.67	0.82	0.21	0.74	0.61	0.27
O2	Effect on local water balance	0.17	0.09	0.72	0.16	0.29	0.29
O3	Effect on local cooling	0.17	0.09	0.07	0.10	0.11	0.04
T1	Uncontrolled flooding	0.30	0.78	0.08	0.33	0.37	0.29
T2	Risk of tree felling	0.16	0.07	0.56	0.33	0.28	0.22
T3	Risk of bank destabilization	0.54	0.15	0.36	0.33	0.35	0.16

### Eighth step: spatializing the SWOT: map aggregation

To explore the sub-objectives, three maps were produced by intersecting ideal beaver habitat suitability with different opportunities or threats.

• The first map, which focused on beaver conservation, intersected beaver needs with low threats.
• The second map, which focused on mitigation of urban heat island effects through increased local water absorption, intersected beaver needs with high levels of O2 and O3, corresponding to areas characterized by low groundwater recharge and high number of tropical nights, respectively. To reflect the dependence of O2 and O3 on damming behavior, only areas with unsuitable water depth were selected.
• The third map, which focused on the increase in rewilded urban green and blue spaces, intersected beaver needs with high levels of O1, representing areas with low supply of green areas.

**Fig. 3:**
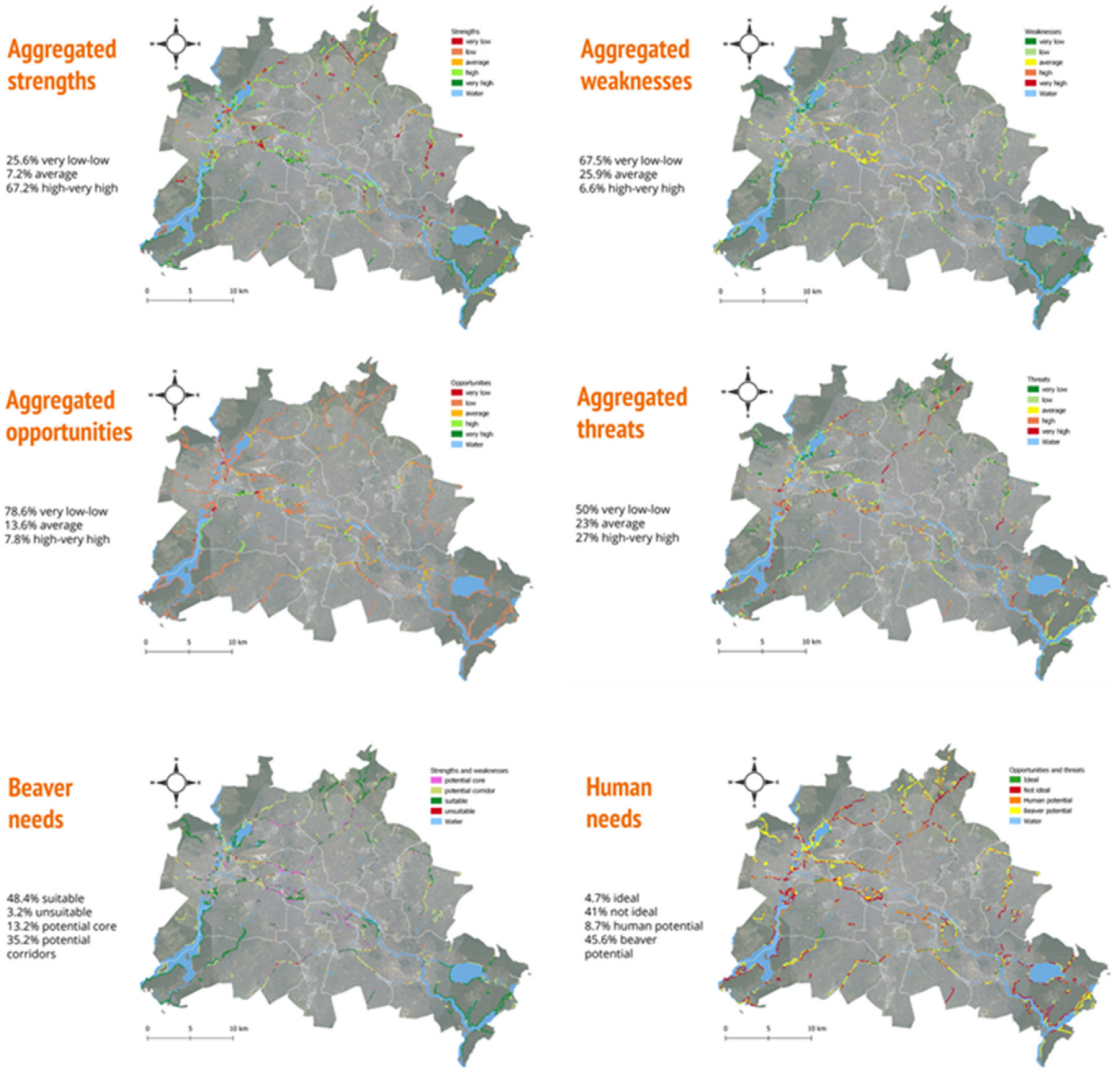
Mapped strengths, weaknesses, opportunities and threats as well as beaver needs (= nature part of the social-ecological system) and human ones (= human part), for the city of Berlin.

In this study, thresholds were determined using equal intervals of the standardised value. For strengths and opportunities, 0–0.5 indicates low suitability and 0.5–1 indicates high suitability, while for weaknesses and threats, 0–0.5 indicates high suitability and 0.5–1 indicates low suitability. Therefore, the ideal suitability of beavers is characterised by high strengths (0.5–1) and low weaknesses (0–0.5), as shown in Fig. 3.

### Ninth step: prioritizing restoration areas

**Fig. 4:**
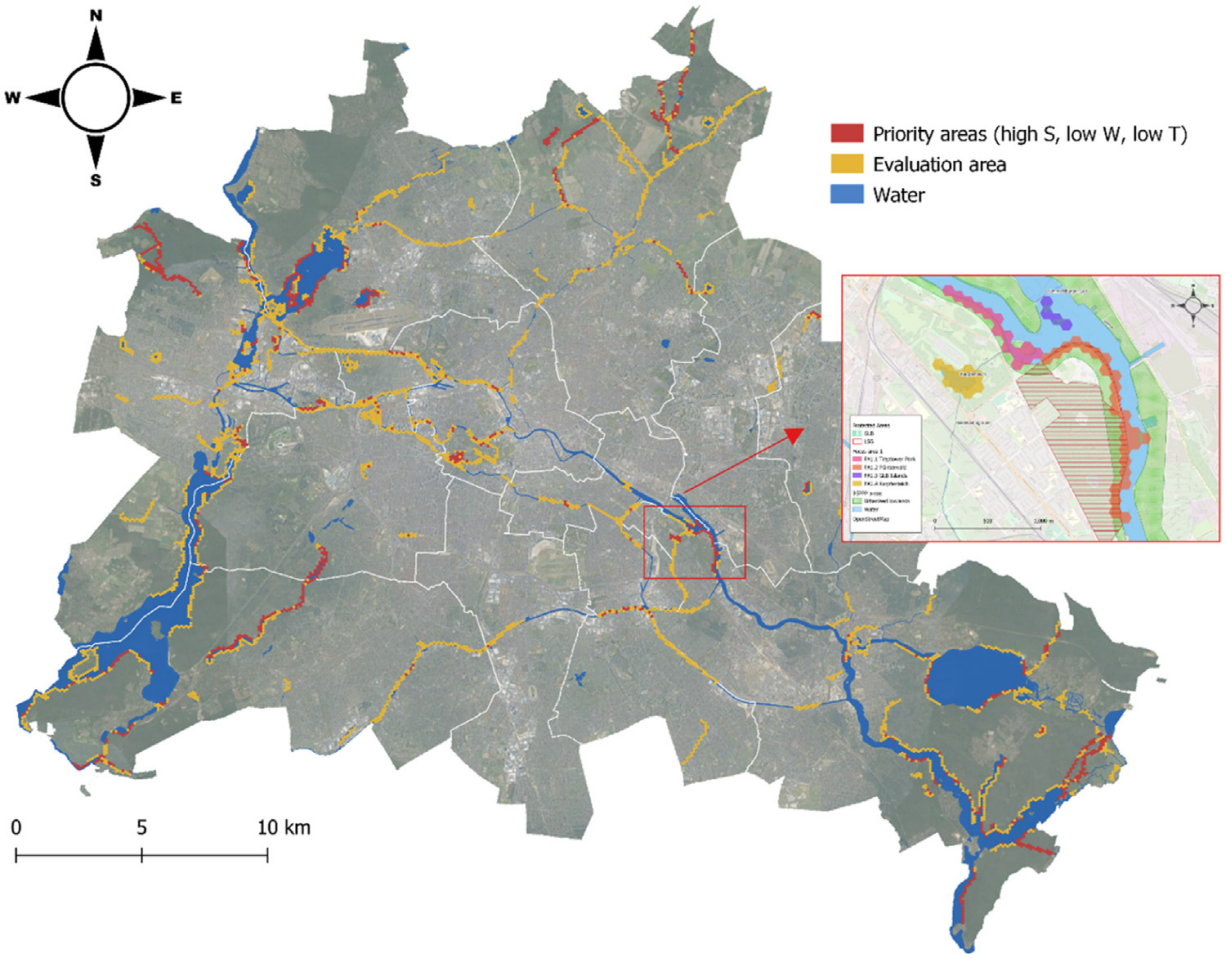
Map for SO1 beaver conservation with zoom on focus area.

**Fig. 5:**
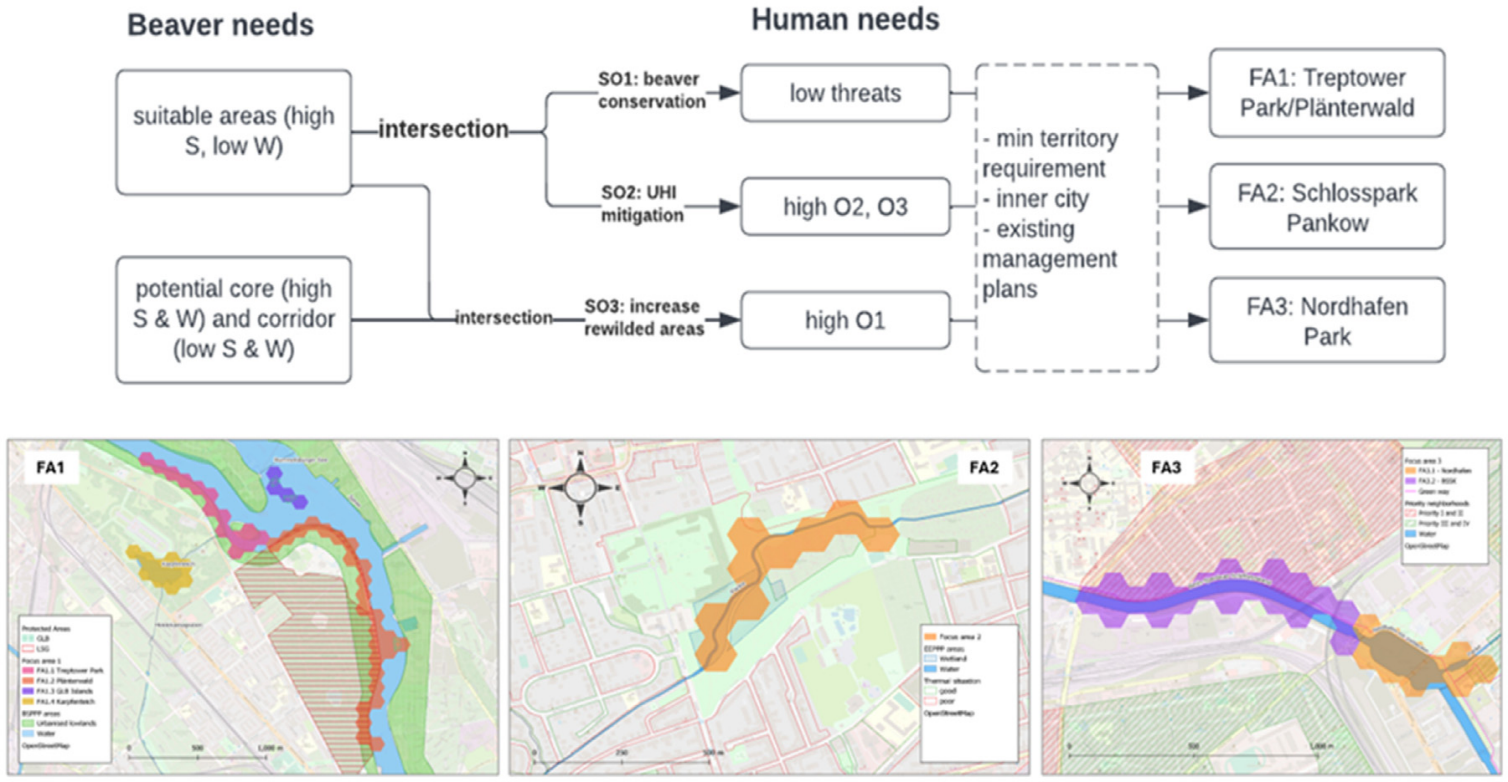
Selection procedure of ‘zoom-in’ test areas.

After aggregating the maps, potential priority restoration areas (focus areas, FAs) were identified based on their ability to fulfil the study objective(s). The largest and most continuous stretch for each sub-objective map was selected as a potential focus area (refer to Fig. 4, Fig. 5). These areas were mostly located on the outskirts of the city, highlighting the difficulty of accommodating non-human species within the urban core. The selection of focus areas was intentionally limited to built-up areas to demonstrate the potential of urban rewilding. The validity of the analyzed data was confirmed through physical site visits.

### Tenth step: recommendations

In the Berlin case study, synergies between beaver engineering activities and current/planned measures were identified. Recommendations were made for creating space for beavers to have a desired impact while reducing potential human-beaver conflicts (refer to Fig. 3, Fig. 4, Fig. 5 for more information).

## Limitations

The final maps provide a preliminary indication on potential areas to consider where the presence of non-human animals may also benefit human communities. Further studies should be conducted to assess the feasibility of the restoration measure as the methodology may over-simplify certain ecological processes. For instance, animal behaviors, which provide co-benefits for humans are dependent on a host of biophysical factors. Hence, assessing opportunities tied to such activities will require an initial evaluation of the likelihood of the animal completing this behavior in the given context. Beavers generally dam when preferred sites are occupied, water depth is unsuitable, sufficient building material is present and river morphology allows damming [23,63]. In this study, only water depth was considered due to lack of resources to complete a more comprehensive assessment. Nevertheless, it is important to note that the uncertainty concerning dam building activities and its effects was captured by the experts, which led to the disproportionate weight of O1 instead of O2 and O3 (damming-related opportunities). This underlines the value of including value based judgments into the evaluation. In order to gain a more reliable evaluation of the ecosystem services provided by beavers in relation to damming, it is necessary to include additional criteria that explain this behavior, particularly in areas where space may be available for beavers to fulfil their role as ecosystem engineers. It is important to note, however, that the positive impacts of beavers extend beyond dam building, and may include impacts on river/channel bank morphology, sedimentation and biodiversity through burrowing [32] or indirect benefits related to tourism or human wellbeing. Further research is required to identify the underlying mechanisms and quantify the impact of these non-damming ecosystem services. This will enable a more comprehensive assessment of the co-benefits considered when this method is applied to beavers.

With the return of the Eurasian beaver in urban ecosystems, this method can guide decision-makers and urban planners on potential rewilded beaver areas in other cities. The proposed set of criteria could be adopted in urban contexts similar to Berlin, a green polycentric and low-density city. A consultation with local experts is still necessary to ensure the indicators remain relevant in the urban context analyzed. Changes in criteria should always follow the complete process of selection described and the balance between SWOT criteria should be kept. The method proposed is also generally applicable to other species. While the 10 steps remain the same, the species-dependent spatial criteria will change.

The MCSDSS comprises the most critical step of the described methodology. It is essential to have a strong scientific basis throughout the entire process since each choice affects the following step and ultimately the analysis results. Furthermore, involving a range of stakeholders is necessary to ensure the broadest support for the course of action. When resources are limited, scientific and participatory guidelines can be quickly sidelined. To strike a balance between the constraints of the modeling process and the closest representation of reality, researchers must carefully evaluate the resulting trade-offs.

The presented method adapts a well-known decision-making framework to the multispecies context, providing much-needed practical guidelines in light of the growing sensitivity towards non-human causes. As previously stated, participation is crucial to the analysis. Therefore, future research could focus on adapting (online) questionnaires/app-based surveys to include typically excluded groups in a low-barrier way. Additionally, the methodology could be further developed to incorporate tools for valuing ecosystem services. This would provide decision-makers with better information to determine the best course of action. Lastly, to account for uncertainties in the decision-making process related to measurement or conceptual errors, a sensitivity analysis could be added to the formal approach to enable the systematic consideration of the manner, in which such errors may affect the final outputs [64].

## Ethics statements

Not applicable

## CRediT authorship contribution statement

**Sophia Rouella Edejer:** Conceptualization, Methodology, Validation, Data curation, Writing – original draft. **Dagmar Haase:** Conceptualization, Writing – review & editing, Supervision. **Matthew Dennis:** Writing – original draft, Writing – review & editing. **Annegret Larsen:** Writing – original draft, Writing – review & editing.

## Declaration of competing interest

The authors declare that they have no known competing financial interests or personal relationships that could have appeared to influence the work reported in this paper.

## Data Availability

Data will be made available on request.

## Appendix Supplementary materials

Image, application 1
