# Supplementary material for: Ten steps towards integrated decision making for ecological restoration in cities: Rewilding the European beaver (Castor fiber) in Berlin, Germany
Source: MethodsX. 2024 Jun 27;13:102827. doi: 10.1016/j.mex.2024.102827 (PMC11262173; doi:10.1016/j.mex.2024.102827)
Supplement: Supplementary file 1 [file mmc1.docx]

**Supplementary material *and/or* additional information [OPTIONAL]**

| Table A1 Description of study criteria | | | | |
| --- | --- | --- | --- | --- |
| Indicator | Proxies | Classes | Standardization | Reference |
| **Strengths:** elements facilitating beaver settlement | | | | |
| **S1** Presence of suitable winter food | Vegetation (ATKIS)  Woods (Environmental Atlas, ATKIS)  Trees (Artenfinder, SenUVK) | 5 discrete classes:   1. **Preferred deciduous**: Alder (*Alnus)*, Ash (*Fraxinus*), Poplar (*Populus)*, Willow (*Salix)* 2. **Other deciduous** 3. **Deciduous and coniferous** 4. **Coniferous** 5. **Other vegetation:** bushes, grass | $x\{\begin{aligned} 1,x=1 \\ 2,x=0.8 \\ 3,x=0.6 \\ 4,x=0.4 \\ 5,x=0 \end{aligned}$ | Beavers are general herbivores, preferring to feed on deciduous trees, especially willow, poplar, alder and ash (Treves et al, 2020). Their diet can also include coniferous trees, as well as herbaceous plants, especially in the summer (Pollock et al, 2015).  Treves et al. (2020) – Species composition of woody vegetation: preferred deciduous (100), deciduous (80), mixed (60), coniferous (40), rest of territory (0)  Adaptation: other vegetation replaces the rest of the territory (scoring 0 as there are no trees but may still be suitable for beavers) |
| **S2** Suitable water level | Stream depth  (Wasserportal, SenUVK)  5m negative buffer | Boolean:   1. **< 50 cm** 2. **≥ 50 cm** | $x\{\begin{aligned} 1,x=0 \\ 2,x=1 \end{aligned}$ | Water depth under 50 cm prevents beaver colonization due to the lack of protection from predators (Maringer & Slotta-Bachmayr, 2006). Beaver sightings in Berlin nevertheless indicate that they are important corridors for dispersal (scoring 0). Water depth is measured within a buffer area of 5m (Pinto et al, 2009).  Maringer & Slotta-Bachmayr, 2006 – Water level: < 50 cm (0), ≥ 50 cm (1) |
| **S3** Natural water network | Structural water quality (Environmental Atlas)  Water constructions (ATKIS, Open Street Map)  Water/wetland type (ATKIS, Environmental Atlas) | 3 discrete classes:   1. **Natural**: unverändert, gering verändert, wetlands 2. **Semi-natural**: deutlich verändert, mäßig verändert, bank reinforcement, lakes, streams 3. **Artificial**: stark verändert, sehr stark verändert, vollständig verändert, bank wall, culverts, locks, dams, piers, marinas, ports, canals, ditches, ponds | $x\{\begin{aligned} 1,x=1 \\ 2,x=0.5 \\ 3,x=0 \end{aligned}$ | Beavers prefer stable natural earth banks protected by dense foliage. Steel and concrete bank walls are unsuitable for beavers (Treves et al., 2020) but beavers may still burrow through bank reinforcements (Lührte & Krauss, 2014). Hydraulic constructions can be obstructed by beaver activities (culverts) as well as present major obstacles (locks, piers, etc.) (Treves et al., 2020). Beavers are opportunistic animals and can also colonize artificial features, such as ditches, canals, and ponds (Pollock et al., 2015).  No precedent exists so the proposed standardization is in the form of gradual equal intervals (expert interview). |
| **Weaknesses:** elements holding back beaver settlement | | | | |
| **W1** Human activity | Land use (Environmental Atlas)  Wetlands (Environmental Atlas, ATKIS) | 5 discrete classes:   1. **Commercial/residential** 2. **Recreational**: Stadtplatz/Promenade, Park/Grünfläche, Sportnutzung, Friedhof 3. **Productive**: Baumschule/Gartenbau, Kleingarten, Ackerland, Grünland 4. **Without specific function**: Brachfläche 5. **Naturalistic**: Wald, wetlands | $x\{\begin{aligned} 1,x=1 \\ 2,x=0.8 \\ 3,x=0.6 \\ 4,x=0.4 \\ 5,x=0.2 \end{aligned}$ | Ideally, beavers would live in riparian forests with minimal disturbance. In a city, such habitat is scarce forcing beavers to adapt and colonize areas such as the Schloßpark Charlottenburg or the Tiergarten. The type of use captures the general level of human activity. Moors and swamps are considered naturalistic as beavers are known to colonize such areas, such as the Tegler Fließ.    Treves et al. (2020) – Function of woody vegetation: naturalistic (100), protective (80), tourist-recreational (60), without specific function (50), productive and protective (40), productive (20), rest of territory (0)  Adaptation: reversed since weaknES rather than a strength. ‘Commercial/residential’ is classified as the most disruptive human activity (scoring 1) followed by ‘Recreational’ in an urban context rather than ‘Productive’. ‘Naturalistic’ scores 0.2 (instead of 0) since there is still human activity. ‘Without specific function’ scores 0.4 (instead of 0.5) as in this context, waste lands have rather less human activity than more. |
| **W2** Human presence at night | Artificial lighting (SenUVK)  Buffer zones: 0-20m, 20-80m, 80-200m | Continuous classes  n_lights^0-20^ * 3 + n_lights^20-80^ * 2 + n_lights^80-200^ | $\frac{x-x^{min}}{x^{max}-x^{min}}$ | Beavers are nocturnal and generally shy animals. They are most active at night when human presence and disturbance are reduced. As central-place foragers, beavers will not venture further than needed from their lodges.  Pachinger & Hulik (1999) – Distance to walkway: 1-20m (1 point), 20-80m (2 points), > 80m (3 points)  Adaptation: distance and number of lights are taken into account to produce continuous classes, where a higher score represents closer and/or more artificial lighting. |
| **W3** Risk of road accidents | Streets (SenUVK) | 3 discrete classes   1. Main roads: StEP classes I, II, III 2. Secondary roads: StEP classes IV, V 3. No roads/risk | $x\{\begin{aligned} 1,x=1 \\ 2,x=0.5 \\ 3,x=0 \end{aligned}$ | According to the Tagesspeigel, the biggest known cause of beaver deaths in Berlin is roadkill (Jacobs, 2021). The busier the street, the bigger the risk.  No precedent exists so the proposed standardization is in the form of gradual equal intervals (expert interview). |
| **Opportunities:** benefits of beaver presence | | | | |
| **O1** Creation of rewilded areas | Supply of green space (Environmental Atlas) | Continuous classes | $1-\frac{x-x^{min}}{x^{max}-x^{min}}$ | Beavers change their environment to more complex, functional and biodiverse areas (Pollock et al, 2015). The creation of green natural spaces can benefit humans in terms of recreation, sport, and well-being. (Gandy & Watts, 2021) |
| **O2** Effect on local water balance | Groundwater recharge (Environmental Atlas) | Continuous classes | $\frac{x-x^{min}}{x^{max}-x^{min}}$ | Through damming, beavers can increase the water table of an area therefore increasing the absorption capacity of said area (Pollock et al., 2015). Such is the case in the Tegler Fließ, where beavers have been present for more than a decade and have contributed to the regulation of the water level, especially during hot summers when rainfall was low (Stork, 2015). |
| **O3** Effect on local cooling | Increase in tropical nights (Environmental Atlas) | Continuous classes | $\frac{x-x^{min}}{x^{max}-x^{min}}$ | Directly linked to the effect on the local water balance is the indirect effect on local cooling of the area. Predictions point towards Berlin getting warmer in the medium and long term, affecting people and environment (SenUVK, n.d.). Beavers can create cooler microclimates through beaver ponds, saturated soils and denser vegetation. |
| **Threats**: negative effects due to beaver presence | | | | |
| **T1** Uncontrolled flooding | Flood risk areas (Environmental Atlas)  Culverts (ATKIS) | 4 discrete classes:   1. **Culverts, High probability** 2. **Medium probability** 3. **Low probability** 4. **Rest of territory**: no data/risk | $x\{\begin{aligned} 1,x=1 \\ 2,x=0.66 \\ 3,x=0.33 \\ 4,x=0 \end{aligned}$ | Beaver flooding occurs when the animals build a partial or complete dam, which creates beaver ponds and/or causes lateral flow of water. In an urban context of narrow channels and high-infrastructure density, this may lead to inundated trees, property, paths and roadways (M. Pollock et al., 2015). Beavers are also known to block culverts as a low-effort alternative to dam building, causing flooding and property damages. Such is the case in the Tiergarten, where beavers have been causing low water levels in some of the much-beloved ponds due to their dams (Bild, 2018).  No precedent exists so the proposed standardization is in the form of gradual equal intervals (expert interview). |
| **T2** Risk of damages through tree felling | Vegetation (ATKIS)  Woods (Environmental Atlas, ATKIS)  Trees (Artenfinder, SenUVK)  Paths (Open Street Map)  Agricultural roads (ATKIS)  Streets (VIZ)  20m buffer zone | 5 discrete classes:   1. **Preferred deciduous**: Alder, Ash, Poplar, Willow 2. **Other deciduous** 3. **Deciduous and coniferous** 4. **Coniferous** 5. **Rest of territory**: outside of 20m buffer, other veg | $x\{\begin{aligned} 1,x=1 \\ 2,x=0.8 \\ 3,x=0.6 \\ 4,x=0.4 \\ 5,x=0 \end{aligned}$ | As was reported by the Morgen Post last year (Morgen Post, 2021), half-felled trees may be a danger for passers-by. According to experts, the danger is assumed to only exist within the immediate vicinity (20m) of water areas.  Standardization identical to S1 – Presence of suitable trees. |
| **T3** Risk of damages through bank destabilization | Paths (Open Street Map)  Agricultural roads (ATKIS)  Streets (VIZ)  4m buffer zone | Boolean:   1. **Risk**: path/road within 4m of water 2. **No risk:** outside of 4m buffer, no path/road, artificial or land area (see S3) | $x\{\begin{aligned} 1,x=0 \\ 2,x=1 \end{aligned}$ | Although fast-moving rivers are considered sub-optimal habitat, beavers may still colonize them if other more suitable territories are lacking as long as the bank allows for the tunnelling of lodges. The burrowing of beavers into banks (1-4 m) may cause bank destabilization and presents a considerable risk for paths or roads whose structural integrity may be compromised by the beaver’s activities (M. Pollock et al., 2015), ultimately leading to injuries or public and private infrastructure damages. This is only evaluated in natural and semi-natural areas (see S3). |

| **Table A2: Metadata** | | | | | | |
| --- | --- | --- | --- | --- | --- | --- |
| **#** | **Data** | **Data Source** | **Data type** | **Last update** | **Retrieved** | **URL** |
| S1 | Woods | Environmental Atlas | Polygon | 2014 | 06.01.2022 | https://fbinter.stadt-berlin.de/fb/index.jsp?loginkey=showMap&mapId=wmsk_05_04forst2014@senstadt |
|  | Citizens’ observations | ArtenAnalyse | Point | 2022 | 22.02.2022 | https://www.berlin.artenanalyse.net/artenanalyse_berlin/ |
|  | Tree Stock | FIS-Broker | Point | 2021 | 21.01.2022 | https://fbinter.stadt-berlin.de/fb/index.jsp?loginkey=zoomStart&mapId=k_wfs_baumbestand@senstadt&bbox=385826,5817832,387563,5818702 |
|  | Vegetation | ATKIS | Polygon | 12.11.2021 | 03.03.2022 | https://fbinter.stadt-berlin.de/fb/index.jsp?loginkey=zoomStart&mapId=atkis_basis_dlm@senstadt&bbox=366009,5810767,402213,5828909 |
|  | Woods | ATKIS | Polygon | 12.11.2021 | 07.01.2022 | https://fbinter.stadt-berlin.de/fb/index.jsp?loginkey=zoomStart&mapId=atkis_basis_dlm@senstadt&bbox=366009,5810767,402213,5828909 |
| S2 | Stream depth | WasserPortal | Excel (point) | Daily | 02.02.2022 | https://wasserportal.berlin.de/messwerte.php?anzeige=tabelle&thema=ws |
|  | Morphology of water bodies | FIS-Broker | WMS | 21.04.2009 | 03.02.2022 | https://fbinter.stadt-berlin.de/fb/index.jsp?loginkey=zoomStart&mapId=gewmor2@senstadt&bbox=399317,5802749,421105,5813668 |
| S3 | Water constructions | ATKIS | Line | 12.11.2021 | 06.01.2022 | https://fbinter.stadt-berlin.de/fb/?loginkey=alphaDataStart&alphaDataId=s_atkis_AX_bauwerkimgewaesserbereich_l@senstadt |
|  | Lock | ATKIS | Polygon | 12.11.2021 | 02.02.2022 | https://fbinter.stadt-berlin.de/fb/?loginkey=alphaDataStart&alphaDataId=s_atkis_AX_bauwerkimgewaesserbereich_l@senstadt |
|  | Water constructions | Open Street Map | Polygon, line, point | Continuously | 21.08.2022 | https://overpass-turbo.eu/  filter for amenity=ferry_terminal, man_made=embankment, marina, pier |
|  | Structural quality | Environmental Atlas | Line | 2011 | 06.01.2022 | https://fbinter.stadt-berlin.de/fb/index.jsp?loginkey=zoomStart&mapId=wmsk_02_06_1_GewStrukGesamt_2011@senstadt&bbox=387403,5818404,395888,5822656 |
| W1 | Function of green and open areas | Environmental Atlas | Polygon | 2020 | 18.02.2022 | https://fbinter.stadt-berlin.de/fb/index.jsp?loginkey=zoomStart&mapId=k06_02_freifl2020@senstadt&bbox=387403,5818404,395888,5822656 |
|  | Moors | Environmental Atlas | Polygon | 2015 | 21.12.2021 | https://fbinter.stadt-berlin.de/fb/index.jsp?loginkey=zoomStart&mapId=wmsk01_19_1moorbodtyp2015@senstadt&bbox=387419,5831380,393307,5834331 |
|  | Swamps | ATKIS | Polygon | 12.11.2021 | 21.12.2021 | https://fbinter.stadt-berlin.de/fb/index.jsp?loginkey=zoomStart&mapId=atkis_basis_dlm@senstadt&bbox=366009,5810767,402213,5828909 |
| W2 | Artificial light | FIS-Broker | Point | 2021 | 14.02.2022 | https://fbinter.stadt-berlin.de/fb/index.jsp?loginkey=zoomStart&mapId=k_oeffbeleucht@senstadt&bbox=388420,5811363,395863,5817495 |
| W3 | Street network | FIS-Broker | Line | 28.02.2022 | 09.08.2022 | https://fbinter.stadt-berlin.de/fb/index.jsp?loginkey=zoomStart&mapId=k_vms_detailnetz_wms_spatial@senstadt&bbox=389232,5819320,394059,5821740 |
|  | Walls | FIS-Broker | Line | 2014 | 10.08.2022 | https://fbinter.stadt-berlin.de/fb/index.jsp?loginkey=zoomStart&mapId=k_StraDa@senstadt&bbox=389101,5824134,389508,5824342 |
| O1 | Supply of green spaces | Environmental Atlas | Polygon | 2013 | 21.12.2021 | https://fbinter.stadt-berlin.de/fb/index.jsp?loginkey=zoomStart&mapId=k09_01_3UGgruen2012@senstadt&bbox=391908,5811330,403201,5816989 |
| O2 | New Groundwater Formation | Environmental Atlas | Polygon | 2017 | 21.12.2021 | https://fbinter.stadt-berlin.de/fb/index.jsp?loginkey=zoomStart&mapId=k02_17gwneu2017@senstadt&bbox=391486,5814828,401509,5819851 |
| O3 | Tropical nights | Environmental Atlas | Polygon | 2015 | 21.02.2022 | https://fbinter.stadt-berlin.de/fb/index.jsp?loginkey=zoomStart&mapId=wmsk_04121_ST2015@senstadt&bbox=387863,5818002,394607,5821450 |
| T1 | Flood Risk Areas | Environmental Atlas | Polygon | 2019 | 21.12.2021 | https://fbinter.stadt-berlin.de/fb/index.jsp?loginkey=zoomStart&mapId=k02_23_4hwrkhigh2019@senstadt&bbox=375395,5818806,382180,5822207 |
|  | Culverts | ATKIS | Line | 12.11.2021 | 06.01.2022 | https://fbinter.stadt-berlin.de/fb/?loginkey=alphaDataStart&alphaDataId=s_atkis_AX_bauwerkimgewaesserbereich_l@senstadt |
| T2 | Path network | ATKIS | Line | 12.11.2021 | 21.12.2021 | https://fbinter.stadt-berlin.de/fb/index.jsp?loginkey=zoomStart&mapId=atkis_basis_dlm@senstadt&bbox=366009,5810767,402213,5828909 |
|  | Street network | FIS-Broker | Line | 28.02.2022 | 09.08.2022 | https://fbinter.stadt-berlin.de/fb/index.jsp?loginkey=zoomStart&mapId=k_vms_detailnetz_wms_spatial@senstadt&bbox=389232,5819320,394059,5821740 |
|  | Agricultural Road network | ATKIS | Line | 12.11.2021 | 21.02.2022 | https://fbinter.stadt-berlin.de/fb/index.jsp?loginkey=zoomStart&mapId=atkis_basis_dlm@senstadt&bbox=366009,5810767,402213,5828909 |
|  | Trees | S1 | Polygon | / | / | / |
| T3 | Path and road network | T2 | Line | / | / | / |
| **Evaluation area** | | | | | | |
|  | State lines | ALKIS | Polygon | 05.08.2022 | 21.08.2022 | https://fbinter.stadt-berlin.de/fb/index.jsp?loginkey=zoomStart&mapId=k_alkis_bezirke@senstadt&bbox=356777,5800624,429185,5836910 |
| Built-up | Land use | Environmental Atlas | Polygon | 2020 | 18.02.2022 | https://fbinter.stadt-berlin.de/fb/index.jsp?loginkey=zoomStart&mapId=k06_01_1realnutz2020@senstadt&bbox=387403,5818404,395888,5822656 |
|  | Building | OSM | Polygon | Continuously | 21.08.2022 | https://overpass-turbo.eu/  filter for landuse=commercial, construction, industrial, residential, retail |
| Water and wetlands | Moors | Environmental Atlas | Polygon | 2015 | 21.12.2021 | https://fbinter.stadt-berlin.de/fb/index.jsp?loginkey=zoomStart&mapId=wmsk01_19_1moorbodtyp2015@senstadt&bbox=387419,5831380,393307,5834331 |
|  | Swamps | ATKIS | Polygon | 12.11.2021 | 21.12.2021 | https://fbinter.stadt-berlin.de/fb/index.jsp?loginkey=zoomStart&mapId=atkis_basis_dlm@senstadt&bbox=366009,5810767,402213,5828909 |
|  | Water map | FIS-Broker | Polygon, line | 01.09.2017 | 22.12.2021 | https://fbinter.stadt-berlin.de/fb/index.jsp?loginkey=zoomStart&mapId=gewkarte@senstadt&bbox=387403,5818404,395888,5822656 |
| **Policy and management plans** | | | | | | |
| LaPro | EEPPP | FIS-Broker | Polygon, line, point | 18.02.2016 | 12.08.2022 | https://fbinter.stadt-berlin.de/fb/index.jsp?loginkey=zoomStart&mapId=lapronatur@senstadt&bbox=386324,5817863,396966,5823196 |
|  | LPP | FIS-Broker | Polygon, line, point | 18.02.2016 | 12.08.2022 | https://fbinter.stadt-berlin.de/fb/index.jsp?loginkey=zoomStart&mapId=laprolabild@senstadt&bbox=386533,5817968,396758,5823092 |
|  | ROSUPP | FIS-Broker | Polygon, line, point | 18.02.2016 | 12.08.2022 | https://fbinter.stadt-berlin.de/fb/index.jsp?loginkey=zoomStart&mapId=laprobiot@senstadt&bbox=387403,5818404,395888,5822656 |
|  | BSPPP | FIS-Broker | Polygon, line, point | 18.02.2016 | 12.08.2022 | https://fbinter.stadt-berlin.de/fb/index.jsp?loginkey=zoomStart&mapId=laproerhol@senstadt&bbox=386533,5817968,396758,5823092 |
|  | Target species | FIS-Broker | WMS | 2009 | 12.08.2022 | https://fbinter.stadt-berlin.de/fb/index.jsp?loginkey=zoomStart&mapId=lapro_aev_ba2015@senstadt&bbox=375463,5820421,381953,5823674 |
|  | Climate model | Environmental Atlas | Polygon, line, point | 2015 | 15.08.2022 | https://fbinter.stadt-berlin.de/fb/index.jsp?loginkey=zoomStart&mapId=wmsk_0411021_planungshin_haupt@senstadt&bbox=389418,5817088,402442,5823615 |
|  | Green ways | FIS-Broker | Line | Yearly (01.05.2022) | 19.08.2022 | https://fbinter.stadt-berlin.de/fb/index.jsp?loginkey=zoomStart&mapId=gruene_wege_wander@senstadt&bbox=380520,5817281,397741,5825911 |
